# Recent Development on the Synthesis Strategies and Mechanisms of Co_3_O_4_-Based Electrocatalysts for Oxygen Evolution Reaction: A Review

**DOI:** 10.3390/molecules30153238

**Published:** 2025-08-01

**Authors:** Yu Liu, Yifan Jia, Hongxing Jia, Liangjuan Gao

**Affiliations:** 1 College of Materials Science and Engineering, Sichuan University, Chengdu 610065, China; liuyu@alu.scu.edu.cn (Y.L.); jiayifan123@stu.scu.edu.cn (Y.J.); 2 College of Materials Science and Engineering, National Engineering Research Center for Magnesium Alloys, Chongqing University, Chongqing 400044, China

**Keywords:** Co_3_O_4_, oxygen evolution reaction (OER), synthesis strategies, mechanism, catalyst

## Abstract

The usage of fossil fuels has resulted in increasingly severe environmental problems, such as climate change, air pollution, water pollution, etc. Hydrogen energy is considered one of the most promising clean energies to replace fossil fuels due to its pollution-free and high-heat properties. However, the oxygen evolution reaction (OER) remains a critical challenge due to its high overpotential and slow kinetics during water electrolysis for hydrogen production. Electrocatalysts play an important role in lowering the overpotential of OER and promoting the kinetics. Co_3_O_4_-based electrocatalysts have emerged as promising candidates for the oxygen evolution reaction (OER) due to their favorable catalytic activity and good compatibility compared with precious metal-based electrocatalysts. This review presents a summary of the recent developments in the synthesis strategies and mechanisms of Co_3_O_4_-based electrocatalysts for the OER. Various synthesis strategies have been explored to control the size, morphology, and composition of Co_3_O_4_ nanoparticles. These strategies enable the fabrication of well-defined nanostructures with enhanced catalytic performance. Additionally, the mechanisms of OER catalysis on Co_3_O_4_-based electrocatalysts have been elucidated. Coordinatively unsaturated sites, synergistic effects with other elements, surface restructuring, and pH dependency have been identified as crucial factors influencing the catalytic activity. The understanding of these mechanisms provides insights into the design and optimization of Co_3_O_4_-based electrocatalysts for efficient OER applications. The recent advancements discussed in this review offer valuable perspectives for researchers working on the development of electrocatalysts for the OER, with the goal of achieving sustainable and efficient energy conversion and storage systems.

## 1. Introduction

In recent years, climate change caused by greenhouse gas emissions due to the combustion of fossil fuels has attracted great attention. To alleviate this problem, there is an urgent need to transition to renewable and environmentally friendly energy sources. Electrochemical energy storage and energy conversion approaches, including fuel cells, CO_2_ reduction, and water electrolysis, have been widely considered, and significant progress has been made recently [1,2,3]. Among the various alternatives, hydrogen is a promising clean energy carrier due to its high energy density and zero carbon emissions. Water electrolysis is a promising technique for hydrogen production that is relatively efficient and cost-effective compared with other techniques, such as steam methane reforming (SMR), partial oxidation of hydrocarbons, biomass gasification, biological production, and so on [4,5,6,7,8]. In addition, the electricity for water electrolysis could be the electricity from solar cells or wind power, which are eco-friendly compared with traditional electricity sources. Furthermore, the oxygen generated during water electrolysis could be used for breathing and combustion reactions. However, the electricity required for water electrolysis is higher than the standard potential of 1.23 V due to the existence of system resistance and polarization barriers (including concentration polarization, ohmic polarization, and activation polarization), which limit the application of water electrolysis [9,10,11,12,13,14]. Therefore, high-performance electrocatalysts are essential for reducing overpotential, especially for oxygen evolution reaction (OER) at the anode and hydrogen evolution reaction (HER) at the cathode under various pH conditions. The OER and HER at different pH levels are shown below:

When pH < 7,

(1) $$\mathrm{Anode}\;\mathrm{(OER)}:\;2H_{2}O\to O_{2}+4H^{+}+4e^{-}$$

(2) $$\mathrm{Cathode}\;\mathrm{(HER)}:\;4H^{+}+4e^{-}\to 2H_{2}$$

When pH > 7,

(3) $$\mathrm{Anode}\;\mathrm{(OER)}:\;4OH^{-}\to O_{2}+2H_{2}O+4e^{-}$$

(4) $$\mathrm{Cathode}\;\mathrm{(HER)}:\;4H_{2}O+4e^{-}\to 2H_{2}+4OH^{-}$$

In the process of water electrolysis, OER is a key step, and it directly affects the efficiency of water electrolysis. The catalytic activity and reaction mechanism of OER are affected by the reaction media of acid and alkali. The OER under acidic (usually using H^+^ as the reactant) and alkaline (usually using OH^−^ as the reactant) environments has manifested different catalytic challenges and opportunities, respectively. Under acidic conditions, OER often requires the use of precious metal catalysts such as iridium (Ir) or platinum (Pt). These precious metals have a low overpotential and are highly efficient in facilitating OER reactions, exhibiting high catalytic activity. However, these catalysts are expensive and are susceptible to corrosion or dissolution. Recently, some precious metal-free electrocatalysts have been studied and reported to be active and persistent against acidic OER. Nonetheless, these precious metal-free electrocatalysts are suffering from severe loss of active sites, typically surviving for only a few hours at 10 mA^−2^ in 0.5 M H_2_SO_4_ [15]. Nowadays, most research focuses on water electrolysis under alkaline conditions. Firstly, water electrolysis under alkaline conditions is more efficient and stable due to the lower overpotential required for the oxygen evolution reaction (OER) at the anode. This results in higher current densities, lower energy consumption, and longer electrode lifetime compared with water electrolysis under acidic conditions. Secondly, the formation of metal oxides during alkaline electrolysis has been extensively studied in recent years, and many transition metal oxides have been found to be effective catalysts for OER in alkaline media. Furthermore, the alkaline electrolyte has a beneficial effect on the catalyst stability since it can effectively buffer the acidic conditions generated during the reaction. This results in a higher stability of the catalyst, which reduces the need for frequent catalyst replacements and improves overall system efficiency. Alkaline water electrolysis has many advantages over acidic water electrolysis in terms of efficiency, catalyst stability, and environmental benefits [16,17,18]. However, alkaline water electrolysis also faces several non-negligible challenges [19]. The slow kinetics of OER and HER under alkaline conditions, especially due to the slow OER, remain a bottleneck for improving system performance. In addition, gas bubble accumulation on electrode surfaces can hinder mass transport and block active sites. Addressing these issues is critical for advancing alkaline electrolysis toward practical and large-scale applications. Therefore, the study of alkaline water electrolysis and the development of efficient and stable catalysts for OER in alkaline media are major focuses of research in the field of materials science.

OER is a critical step in water electrolysis, which involves the production of oxygen gas at the anode. One of the major challenges in developing OER catalysts is the high energy required for the OER reaction, which leads to significant energy losses and reduces the efficiency of the water electrolysis process. Efficient OER catalysts should have the characteristic of lowering the overpotential needed to drive the OER reaction at the anode side. Such a catalyst could help reduce energy losses during the reaction and increase the efficiency of the electrolysis process. Moreover, the OER catalysts should have a high catalytic activity, which is the ability to accelerate the reaction rate, and be stable under the harsh conditions of the water electrolysis process [20,21]. In recent years, many different types of OER catalysts have been developed, including metal oxides, perovskites, and molecular catalysts. Metal oxides, in particular, have been extensively studied and have shown promising potential as OER catalysts in alkaline media. Several transition metal oxides, such as RuO_2_ [22,23], IrO_2_ [23,24], CoO_x_ [25,26,27], NiO_x_ [28,29,30], and FeO_x_ [31,32], have been identified as highly active and stable OER catalysts in alkaline conditions.

Another major challenge in developing efficient OER catalysts is their limited stability over long-term operation, which might lead to a decrease in catalytic activity and efficiency of the electrolysis process. The stability of the catalysts is affected by several factors, including surface morphology, crystalline structure, and the presence of impurities. The use of advanced characterization techniques, such as high-resolution microscopy and spectroscopy [21,22,33], has enabled researchers to understand the fundamental processes underlying the OER reaction and to design more stable catalysts. In addition to developing efficient and stable OER catalysts, the integration of these catalysts into water electrolysis systems is also a critical area of research. The design of the electrode and the optimization of the catalyst loading and distribution on the electrode surface can significantly affect the efficiency and performance of the water electrolysis process.

Although other cobalt-based materials such as Metal Organic Frameworks (MOFs), Metal-Organic Gels (MOGs), Polyoxometalates (POMs), and cobalt-doped porous carbons have also been studied for water-splitting applications, they often suffer from poor stability, limited conductivity, or ambiguous active sites. In contrast, Co_3_O_4_ offers structural stability, mixed-valence states, and excellent electrochemical durability, which has shown excellent catalytic activity and stability under alkaline conditions [34,35,36,37,38]. The unique crystal structure of Co_3_O_4_, which consists of both tetrahedral and octahedral Co sites, provides a high density of active sites for the OER reaction, resulting in high catalytic activity [39,40]. The design and synthesis of Co_3_O_4_-based catalysts for OER in alkaline conditions require a comprehensive understanding of the structure–activity relationship and the factors that affect the catalytic activity and stability. The use of advanced characterization techniques, such as X-ray diffraction, transmission electron microscopy, and X-ray photoelectron spectroscopy, can provide insight into the crystal structure, surface morphology, and chemical composition and state of the catalysts [33,41,42,43]. These techniques can also help identify the factors that affect the catalytic activity and stability of Co_3_O_4_-based catalysts, such as the particle size, the surface area, and the surface chemistry. Recent studies have focused on improving the OER performance of Co_3_O_4_-based catalysts by modifying their composition and structure. Doping Co_3_O_4_ with other elements, such as Mn [44,45], Ni [29,46], and Ce [47,48], can modify the electronic structure and surface chemistry of the catalyst, leading to improved OER activity and stability. Furthermore, the integration of Co_3_O_4_-based catalysts with other materials, such as carbon nanotubes, graphene, and metal substrates, can further enhance the OER performance by improving the electronic and structural properties of the catalysts [49,50,51].

Despite promising catalytic results having been obtained with Co_3_O_4_-based catalysts, there are still several challenges that need to be addressed. One of the main challenges is the poor conductivity of Co_3_O_4_, which can limit the transport of charge carriers and affect the OER performance. Therefore, the integration of Co_3_O_4_-based catalysts with highly conductive materials is critical to improve their catalytic performance. Another challenge is the limited stability of Co_3_O_4_-based catalysts under high OER potentials, which can result in degradation and reduced catalytic activity. Therefore, the development of methods to stabilize Co_3_O_4_-based catalysts and prevent degradation under harsh conditions is an important area of research [39,52]. Thus, in this review, we introduce the synthesis strategies and mechanisms of Co_3_O_4_-based OER catalysts and suggest the future aspects for this topic.

## 2. External Catalytic Performance

As we know, catalysts play a critical role in many chemical reactions. Even small changes to the catalyst’s structure or composition can have a significant impact on its performance. Therefore, current research efforts are focused on improving catalyst performance through two approaches: improving the external catalytic performance and enhancing the intrinsic activity of catalysts. External catalytic performance is largely determined by the active surface area and number of active sites. Generally, techniques of adding porosity and using nanoparticles are used to increase the surface area, while doping or modifying the surface of the catalyst is used to increase the number of active sites. In addition, optimizing the material transfer channel can enhance the mass transfer of reactants and products to and from the catalyst surface, leading to an improvement in reaction rates and efficiency. Intrinsic activity can be enhanced by optimizing the catalyst’s composition, structure, and surface chemistry [53,54]. This can improve the catalyst’s selectivity and reaction rate. Researchers are continually exploring new solutions to improve catalyst performance through these approaches.

### 2.1. Active Surface Areas and Active Sites Enhancements

#### 2.1.1. Morphology Engineering

Nano-sized catalysts have been shown to increase the active area of reactions effectively, thereby increasing the number of active sites. Based on this, nano-sized morphology engineering can further improve the performance of OER [8,34,55]. In recent years, researchers have prepared Co_3_O_4_-based materials with various morphologies, such as nanoneedles [56,57], nanowires [58,59], nanofilms [60,61,62,63], nanocages [64,65], and nanocubes [66].

**Figure 1:**
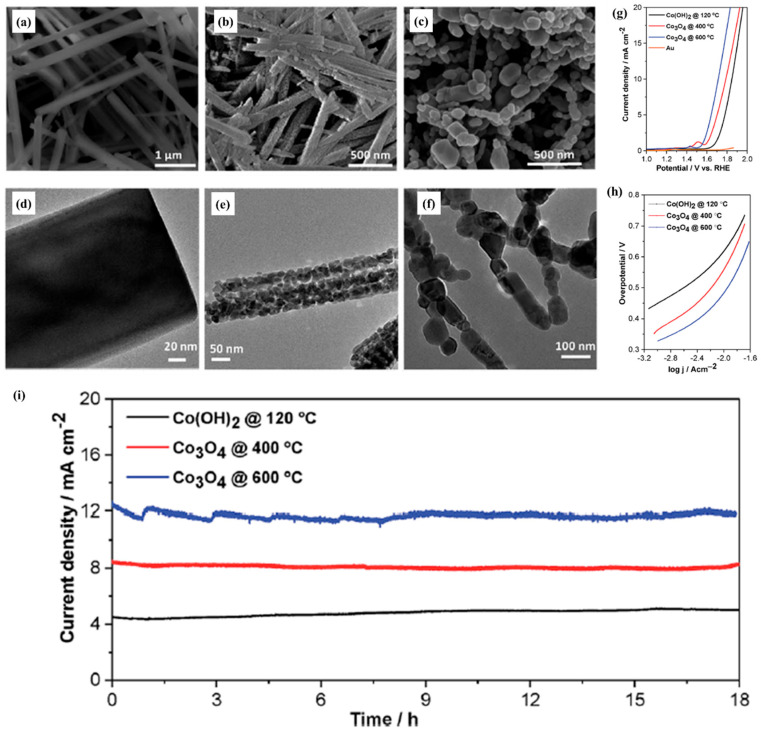
FE-SEM and HR-TEM images of Co(OH)_2_ @ 120 °C (**a**,**d**), Co_3_O_4_@ 400 °C (**b**,**e**), and Co_3_O_4_ @ 600 °C (**c**,**f**) electrocatalysts. (**g**) Linear sweep voltammogram at 1 mV s^−1^. (**h**) Tafel plots and (**i**) time-dependent current density curves of the catalysts under static overpotential of 1.76 V vs. RHE for 18 h in an aqueous solution of 0.1 M KOH (pH 13) (Reproduced from ref. [67] with permission from RSC).

Tao et al. [57] synthesized nanoneedles with different morphologies on Ti mesh through hydrothermal and calcination methods. The catalytic performance of Co_3_O_4_@Ti-05 and Co_3_O_4_@Ti-20 was inferior to that of Co_3_O_4_@Ti-15 due to the synthesis of too little and too much Co(NO_3_)_2_·6H_2_O in the raw material. Scanning electron microscopy (SEM) characterization revealed that the grassy nanoneedles of Co_3_O_4_@Ti-15 almost perfectly cover the Ti mesh without redundant structures, making full use of the active sites of Co_3_O_4_ nanoneedles. In contrast, Co_3_O_4_@Ti-20 generated many chestnuts on the top of the nanoneedles, making it difficult to expose the active sites, fully demonstrating the importance of the increase of active surface area and active sites for the catalytic performance of OER. The catalyst treatment temperature might change the morphology of the catalyst and thus the performance of the catalyst. Dhawale et al. [67] synthesized Co(OH)_2_ nanorods that were converted into Co_3_O_4_ nanorods by subsequent high-temperature treatment. In the process of high-temperature treatment, cetyltrimethylammonium bromide (CTAB) surfactant transformed the nanorods into interconnected porous structures through volume shrinkage and decomposition. Subsequent electrochemical experiments demonstrated that these porous structures provided a large surface area and enriched the active boundary sites of Co_3_O_4_ (see Figure 1a–f). By comparison, the performance of the catalyst was improved with increasing treatment temperature (see Figure 1g–i).

**Figure 2:**
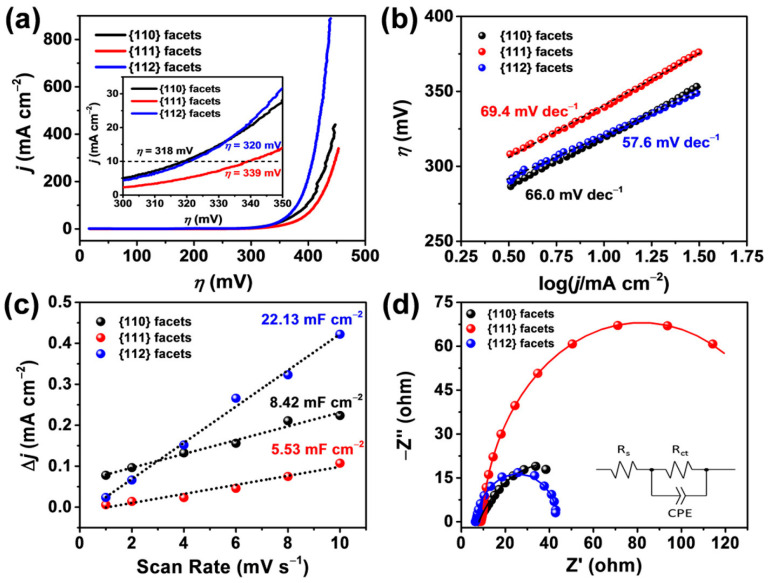
Electrochemical activity comparison of different facets exposed Co_3_O_4_ nanosheets (**a**) LSV curves with a scan rate of 5 mV s^−1^ in 1 M KOH solution; inset is the enlarged version with an overpotential window from 300 to 350 mV. (**b**) Tafel plots and corresponding linear fittings. (**c**) Differences in current density variation at a potential of 0.225 V (vs. SCE) plotted against scan rate fitted to a linear regression, enabling the estimation of C_dl_. (**d**) Nyquist plots obtained from the electrochemical impedance spectroscopy measurements at 1.55 V (vs. RHE) with a frequency range of 0.01 to 3 × 10^5^ Hz (R_s_ means resistance caused by electrical conductivity in electrolyte solutions, R_ct_ means the resistance in the charge transfer process between the electrode surface and the solution, and CPE means constant phase element and models the complex behavior at the interface between electrodes and electrolytes.) (Reproduced from ref. [70] with permission from ACS).

In nanosheet materials, the exposed crystal faces have a great influence on the adsorption and desorption characteristics of the reactants, intermediates, and products. In general, exposed faces with high indices of crystal faces can provide more favorable catalytic surface atomic structures [68,69]. Therefore, Wei et al. [70] prepared three kinds of Co_3_O_4_ exposed by NaBH_4_-reduced crystal surfaces by different methods: {110}, {111}, and {112}. {112} high-index facets had the best catalytic performance, attributed to the largest proportion of Co^2+^/Co^3+^, which provided more active sites for OER (Figure 2a–d). It is not only the above study that suggests the superiority of high-index facets. Zheng et al. [63] prepared Co_3_O_4_ with edge-enriched {111} facets by calcining α-Co(OH)_2_ nanoparticles in air, generating more active sites and atom-step densities than Co_3_O_4_ with poor-edge {111} facets and thus enhancing the efficiency of OER.

In addition to conventional 1D and 2D morphologies, many researchers have also tried to synthesize more complex morphologies to promote the catalytic activity of Co_3_O_4_. Common morphologies include sea urchin-shaped structures [71], flower-shaped structures [72], core-shell structures [31,66,73] and so on. Wang et al. [71] prepared Co_3_O_4_ in the form of flower-like and urchin-like structures on Ni foam (NF) using a simple hydrothermal method followed by high-temperature treatment. The generation of these two morphologies increases the specific surface area of the Co_3_O_4_ catalyst, exposing more active sites on the surface, which is conducive to contact with the electrolyte. The exposed active sites enhance the conductivity of the sample and the capturing ability of the surface hydroxyl group. Therefore, it shows excellent performance, requiring only 327 mV of overpotential to drive a current density of 20 mA cm^−2^.

**Figure 3:**
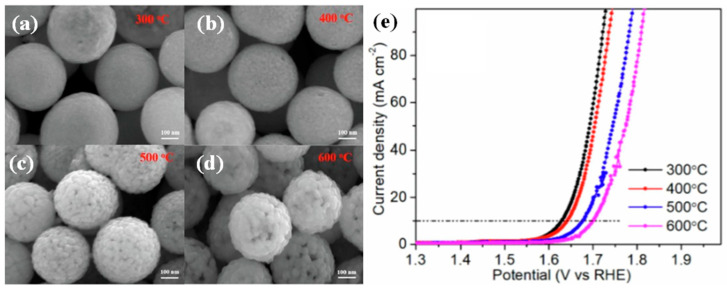
SEM images of Co_3_O_4_ nano-microspheres prepared at different temperatures: (**a**) 300 °C, (**b**) 400 °C, (**c**) 500 °C, (**d**) 600 °C, and (**e**) LSV of OER for Co_3_O_4_ nano-microspheres prepared at different temperatures in 1 M KOH. (Reproduced from ref. [74] with permission from Elsevier).

In order to compare the influence of Co_3_O_4_ particles with different sizes on the catalytic performance of OER, Feng et al. [74] synthesized Co_3_O_4_ nanospheres composed of different particle sizes by a two-step method involving solvothermal and annealing. Annealing temperature is the key to controlling the particle size of Co_3_O_4_. Figure 3a–d shows the SEM images of Co_3_O_4_ nano-microspheres prepared at different temperatures. With the increase in annealing temperature, the size of nanoparticles increases gradually. This phenomenon could be attributed to the increase of diffusion and mass transfer with the increase of annealing temperature, resulting in the neighboring small nanoparticles being fused together and a gradual increase in the size of nanoparticles. In the subsequent electrochemical tests (as shown in Figure 3e), the Co_3_O_4_ nanospheres annealed at 300 °C exhibited the best OER performance, and the catalytic performance of each microsphere decreased gradually with the increase of annealing temperature, demonstrating that the size of the catalyst has a significant influence on OER performance. As can be seen from the surface effect of nanomaterials, small-sized nanoparticles can provide a larger surface area, so the nano-microspheres composed of small particles have more surface area to contact with the electrolyte, thus providing more sites for the adsorption of OH^−^.

Table 1 summarizes the relationship between the structure and performance of Co_3_O_4_-based catalysts and currently commercialized catalysts. Compared with other commercially available OER catalysts, such as Ru or Ir, Co_3_O_4_-based catalysts offer a relatively cost-effective and abundant alternative. However, the presence of precious metals increases the conductivity and thus the catalytic activity of the catalyst, and the stability is correspondingly improved, making it play an important role in the field of commercial OER, but the catalysts based on Co_3_O_4_ show great promise due to their high compatibility, high stability, and potential to improve performance through morphological engineering and doping strategies. In summary, the large specific surface area of nanomaterials provides abundant active surface area and active sites for Co_3_O_4_-based catalysts, and the nanostructures can be interconnected to form porous structures through surfactants, enriching their active boundary sites. By designing a Co_3_O_4_ catalyst with a high index plane, the ratio of Co^2+^/Co^3+^ was maximized, which provided more active sites for OER. In addition, the complex design of the morphology can also improve the catalytic performance of the Co_3_O_4_ catalyst, which has been demonstrated in other metal oxide catalysts used to improve the performance of OER [75,76]. It can be seen from the above results that the catalytic surface area and the number of active sites can be improved by preparing catalysts with specific morphologies and sizes. Therefore, future studies should try to combine the two aspects. For example, it would be promising to reduce the volume of each Co_3_O_4_ “sea urchin” and increase their amounts during the morphology control, which might provide a larger surface area and plenty of active sites and thus improve the OER performance. At the same time, if the catalyst with high-index facets and as small a size as possible could be prepared by a specific method during the synthesis, it might also exhibit superior catalytic performance.

#### 2.1.2. Catalyst Anchoring

**Figure 4:**
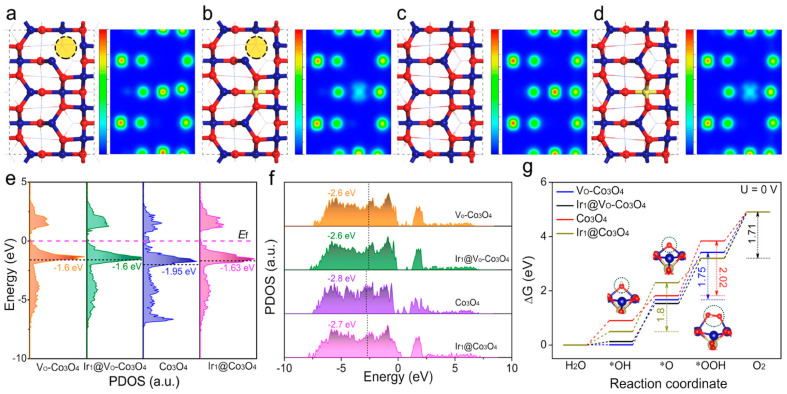
DFT calculations. Structural models and the corresponding charge density distribution plots on the (110) plane of (**a**) Vo–Co_3_O_4_, (**b**) Ir_1_@Vo–Co_3_O_4_, (**c**) Co_3_O_4_, and (**d**) Ir_1_@Co_3_O_4_. Blue ball: Co atom; red ball: O atom; gold ball: Ir atom. The scale bar is from min: 0 (bottom) to max: 1.4 (top). (**e**) The d-band center of Vo–Co_3_O_4_, Ir_1_@Vo–Co_3_O_4_, Co_3_O_4_, and Ir_1_@Co_3_O_4_. (*E*_f_ means Fermi level.) (**f**) O 2p-band center of Vo–Co_3_O_4_, Ir_1_@Vo–Co_3_O_4_, Co_3_O_4_, and Ir_1_@Co_3_O_4_. (**g**) Calculated Gibbs free-energy diagrams for OER on Co sites on the (110) plane of Vo–Co_3_O_4_, Ir_1_@Vo–Co_3_O_4_, Co_3_O_4_, and Ir_1_@Co_3_O_4_. (U means the applied potential.) (Reproduced from ref. [88] with permission from ACS).

The durability of catalysts has always been one of the challenges for commercialization [86]. There are two main factors that affect the durability of the catalyst. Firstly, large numbers of bubbles generated with the increase of operation time might cause the catalysts to gradually fall off the electrode. Secondly, the extreme solution environment might cause the transformation of the catalyst structure and thus reduce the number of catalytic sites [87]. Therefore, the protection of the catalyst is also extremely important, and its essence is to protect the catalytically active area and the number of active sites from being reduced. Chen et al. [88] obtained the mesoporous Ir_x_@Co_3_O_4_ by thermal decomposition of Ir-ZIF-67. The mesoporous structure facilitates the exposure of active sites and transport of bubbles produced during OER. At the same time, long-term in situ Raman spectroscopy was performed on the catalyst at an overpotential of 300 mV, and the test results showed that the presence of Ir single atoms (SAs) inhibited the structural reconfiguration of Co_3_O_4_ during the catalytic process and improved the durability of the catalyst significantly. In addition, anchored Ir SAs can transfer electrons from Co to Ir, thus producing electron-poor Co active sites and electron-rich Ir SAs, improving the catalytic activity and long-term stability of OER (Figure 4a–g). In addition to the above studies, Dai et al. [89] have also prepared catalysts with Ir uniformly anchored to Co_3_O_4_. During the preparation process, the slightly acidic environment provided by the IrCl_4_ solution can promote the dissolution of Co^2+^ in CoCO_3_ and promote the anchoring of Ir. Through simple sintering, Ir@CoCO_3_ can be converted to Ir@Co_3_O_4_. It is worth mentioning that the Ir content of the catalyst is only about 1.4 wt%. This simple and economical production process can provide potential support for industrial mass production. Ir@Co_3_O_4_ can be maintained at a current density of 10 mA cm^−2^ for 120 h at a required potential increase of only 67 mV, which is more stable than that of most catalytic materials. Various characterizations after stability testing show that the structure of Ir@Co_3_O_4_ remains stable, and the crystal structure of Co_3_O_4_ can be maintained by Ir clusters, thereby slowing down the process of its amorphous state, which may be an important reason for the maintenance of the catalytic stability of Ir@Co_3_O_4_.

Besides maintaining catalytic efficiency by preserving the crystal structure of the catalyst itself, protecting the catalyst from falling off the electrode has become one of the top priorities. Therefore, in situ preparation of catalysts on various electrode substrates has been widely studied. The most common substrate for the in situ growth of alkaline OER catalysts is nickel foam, which can provide a large number of growth sites for catalysts and improve the catalytic efficiency per unit area due to its unique three-dimensional network structure [90]. In order to study the influence of the self-supported substrate on the catalyst, Yu et al. [91] prepared Co_3_O_4_ nanosheet Co_3_O_4_ NS/NF by in situ growth on nickel foam through a simple hydrothermal method without high-temperature treatment and prepared Co_3_O_4_ without nickel foam support under the same conditions for comparison. SEM images showed that the 3D structure of nickel foam provided a large area for Co_3_O_4_ nanosheets to be uniformly anchored on it, reducing the aggregation of catalysts and exposing the active sites of the catalysts in large quantities. The comparison of electrochemically active surface area (ECSA) between Co_3_O_4_ NS/NF and Co_3_O_4_ confirms that the former has a higher specific surface area. Additionally, electrochemical impedance spectroscopy (EIS) shows that Co_3_O_4_ NS/NF has almost no diffusion limitation, which is attributed to its in situ growth on NF. This results in a low resistance that provides a basis for rapid migration of electrolyte ions. In stability tests, Co_3_O_4_ NS/NF demonstrates superior performance compared with Co_3_O_4_. The in situ growth process leads to continuous and stable gas precipitation, which makes the catalyst less susceptible to bubbles and falling off the substrate. Furthermore, NF enhances the charge transfer between Co_3_O_4_ nanosheets, reducing the influence of catalyst poisoning on the performance of OER. However, the use of NF is not favorable for the commercialization of catalysts [92], since it is a solid bulk material; this reduces its flexibility for integration in the design and large-scale automated manufacturing process, and it might be better if the in situ growth and anchored substrate were not a large solid like NF.

### 2.2. Material Transport Channels

#### 2.2.1. Catalyst Structure for Material Transfer Control

Aspects of the Co_3_O_4_ catalyst that require improvement to increase its oxygen adsorption/desorption efficiency include the need for less energy to avoid poisoning, which is due to the easy adsorption of intermediates and difficult desorption of final products [39]. Moreover, under longer catalytic time and larger catalytic power, the oxygen adsorption/desorption efficiency should also be enhanced. In response to this need, Chutia et al. [93] prepared octahedral Co_3_O_4_ (Oct-Co_3_O_4_/C) with a mesoporous structure and a large specific surface area on C using a two-step method of solvothermal and calcination. These two characteristics allow for the exposure of active sites. Furthermore, the size and shape effects lead to the formation of more oxygen vacancies on the surface of Oct-Co_3_O_4_/C compared with ordinary Co_3_O_4_ catalysts, which help activate the adsorption of O_2_ and improve the mobility of surface O_2_, thus promoting O_2_ desorption.

Efforts to improve the efficiency with which oxygen is removed from the catalyst itself are important, but it is also essential to improve the efficiency with which oxygen is transferred from the electrode to the electrolyte. For example, Liu et al. [94] directly grew Co_3_O_4_ mesoporous nanorods on nickel foam by hydrothermal methods, which not only promoted the efficient contact between the electrode interface and electrolyte but also showed excellent durability in catalytic durability experiments. The strong contact between Co_3_O_4_ nanorods and nickel foam and the three-dimensional porous structure facilitate the transmission and release of oxygen, thereby reducing the “poisoning” of the catalyst.

**Figure 5:**
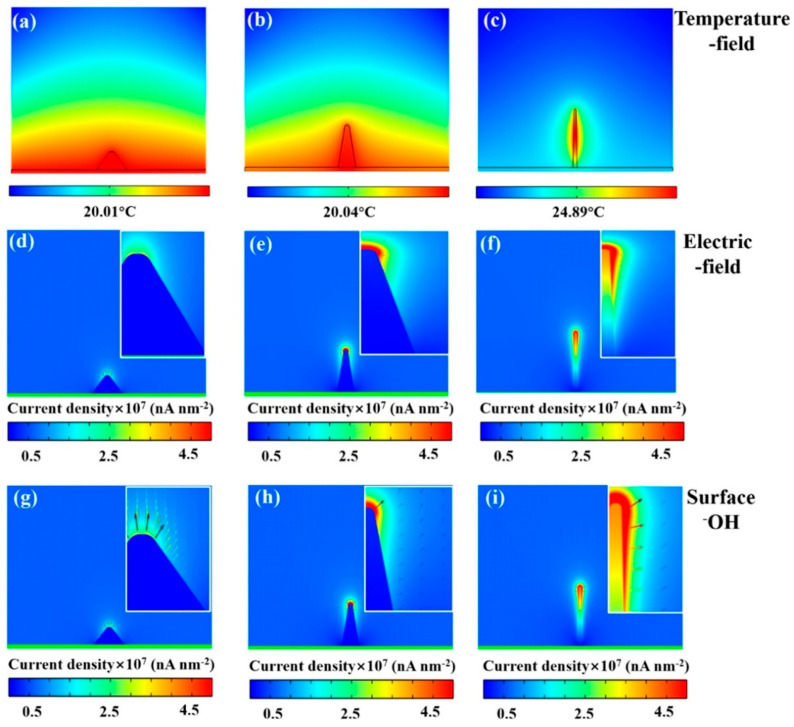
Simulation results with different height–diameter ratios (5:1, 8:1, and 10:1). (**a**–**c**) temperature distribution on the electrode surface. (**d**–**f**) charge distribution and (**g**–**i**) –OH density at surface and movement trend of –OH (Reproduced from ref. [95] with permission from Elsevier).

In addition to transferring oxygen, the transport process of other intermediates is also crucial, and improving the transport efficiency of these products will have a significant impact on the efficiency of OER catalysis. For example, increasing the contact and transmission rate between OH^−^ and the electrode in alkaline electrolyte can improve the reaction speed. To achieve this goal, Zhang et al. [95] regulated the height–diameter ratio of Co_3_O_4_ nanowires through hydrothermal synthesis by adding different proportions of NH_4_F to the raw material. Through the establishment of the catalyst model, it was verified that Co_3_O_4_ nanowires with a height–diameter ratio of 10:1 speed up the transfer of electrons and enhance the “interfacial Joule heating effect”, which heats up the tip of the nanowires, thus increasing the rate constant and diffusion coefficients of H_2_O and OH^−^ within the boundary layer. The tip of the nanowire also has a stronger local electric field in catalysis that promotes the transport of OH^−^, thus increasing the rate of OER reaction (Figure 5a–i), so that it increases the rate constant and diffusion coefficients of H_2_O and OH^−^ within the boundary layer. Additionally, the nanowire tip generated a stronger local electric field during catalysis, promoting the transport of OH^−^ and ultimately increasing the OER reaction rate. We believe that this kind of modeling study is of great significance for studying the catalytic mechanism of catalysts, and perhaps many studies on the catalytic performance of morphologically controlled catalysts can also be started from this aspect.

#### 2.2.2. Combination with Conductive Materials to Reduce Resistance

Research has shown that the inherent conductivity of Co_3_O_4_ is limited due to its crystal structure, making it less effective as a catalyst for OER [96,97]. To address this limitation, combining Co_3_O_4_ with other conductive materials, such as carbon and two-dimensional transition metal carbides (2D MXenes), has become a solution [98]. Carbon is widely used because it is an inexpensive material with good conductivity [87,99,100,101,102]. In one study, Yang et al. [103] pyrolyzed ZIF-67 to obtain a flower-like nitrogen-doped carbon scaffold in situ wrapped by Co_3_O_4_ nanoparticles (M-Co_3_O_4_/NPC). The OER catalytic test on the catalyst showed that it could drive the current density of 10 mA cm^−2^ at an overpotential of 302 mV, and the Tafel slope was as low as 84 mV dec^−1^. Such excellent electrochemical performance is attributed to the fact that the carbon scaffold provided a channel for contact between the electrolyte and catalyst, providing more active sites, while the nitrogen-doped carbon scaffold’s excellent electrical conductivity facilitated electron transmission, contributing to the transfer of charge between the catalyst and electrolyte. Stability tests showed a current attenuation of only 5.3% after 10 h, indicating the essential role of carbon support for Co_3_O_4_.

**Figure 6:**
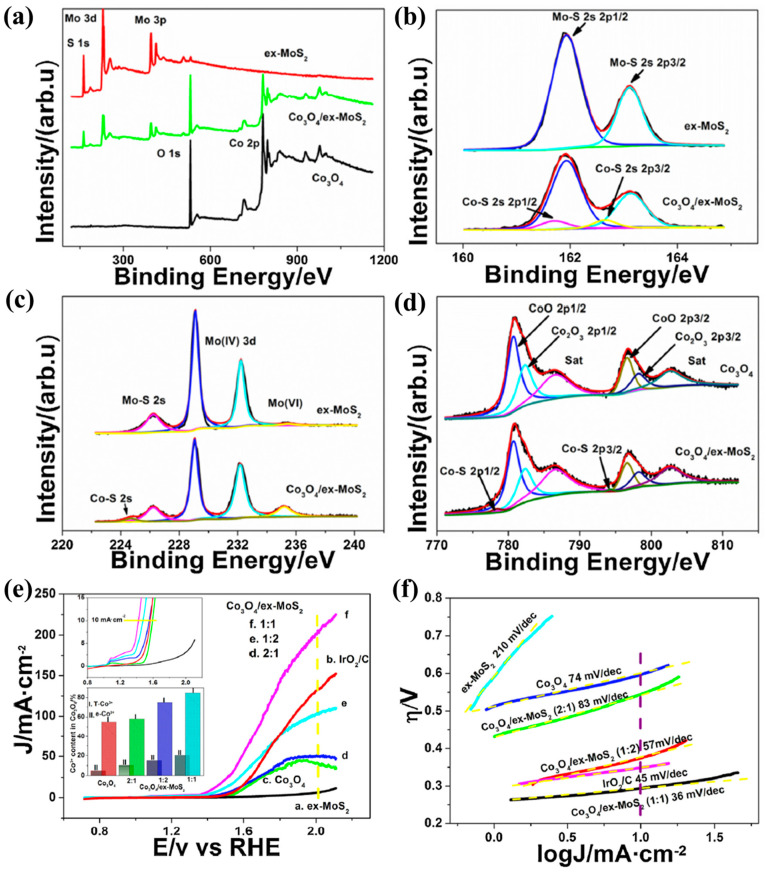
XPS survey spectra (**a**) of ex-MoS_2_, Co_3_O_4_, and Co_3_O_4_/ex-MoS_2_ hybrids (1:1); (**b**) S 1s core level spectra of ex-MoS_2_ and Co_3_O_4_/ex-MoS_2_ hybrids; (**c**) Mo 3d core level spectra of ex-MoS_2_ and Co_3_O_4_/ex-MoS_2_ hybrids; and (**d**) Co 2p core level spectra of Co_3_O_4_ and Co_3_O_4_/ex-MoS_2_ hybrids. (**e**) LSV curves of ex-MoS_2_, Co_3_O_4_, IrO_2_/C, and Co_3_O_4_/ex-MoS_2_ hybrids with different Co_3_O_4_/MoS_2_ molar ratio in 0.1 M KOH solution at the scan rate of 10 mV s^−1^. Inset: their enlarged part of LSV under low current density and Co^3+^ content in Co_3_O_4_ matrix for Co_3_O_4_ and Co_3_O_4_/ex-MoS_2_ hybrids. (**f**) Tafel plots of ex-MoS_2_, Co_3_O_4_, IrO_2_/C, and Co_3_O_4_/ex-MoS_2_ hybrids with different Co_3_O_4_/MoS_2_ molar ratios in 0.1 M KOH solution. (Reproduced from ref. [106] with permission from Elsevier).

However, carbon can be corroded and oxidized during electrocatalysis, particularly when exposed to high alkaline or acidic potential, which can reduce electrical conductivity and adversely affect catalytic performance [104,105]. To address this, Wang et al. [106] prepared ultra-thin Co_3_O_4_ nanosheets grown on MoS_2_ by chemical exfoliation of MoS_2_ and in situ generation of Co(OH)_2_ on MoS_2_, followed by annealing. XPS spectra clearly show the presence of Mo, S, Co, and O in Co_3_O_4_/ex-MoS_2_, and the presence of Mo–S bonding in ex-MoS_2_ to Co–S bonding in Co_3_O_4_/ex-MoS_2_ means the formation of Co–S during Co_3_O_4_ deposition. In Co_3_O_4_/ex-MoS_2_, the new peak of Mo^6+^ at 236.4 eV becomes more prominent due to the oxidation of MoS_2_ during solution stripping and Co_3_O_4_ deposition. The formation of Co^2+^ and Co^3+^ species indicates the presence of Co_3_O_4_ and Co–S bonding, confirming that Co has been integrated into the MoS_2_ structure. Co_3_O_4_/ex-MoS_2_ showed a low overpotential and exaggerated Tafel slope of only 36 mV dec^−1^ in the OER catalytic test. Combined with the analysis of EIS, the catalytic activity of ex-MoS_2_ was not good, but the conductivity was better than that of Co_3_O_4_, indicating that Co_3_O_4_ was the main catalytically active material. However, the presence of ex-MoS_2_ greatly increases the electron transfer rate of Co_3_O_4_ during the catalytic process and improves the catalytic activity. Additionally, the presence of MoS_2_ strengthened the stability of the catalyst due to the formation of possible Co–S bonds, which exhibit high anti-decomposition, anti-corrosion, and anti-dissolution stability in the catalytic process of high electric potential (Figure 6a–f). In addition to MoS_2_, 2D MXenes with superior electrical conductivity can also serve as conductive support materials for Co_3_O_4_ [107,108,109]. Lu et al. [110] prepared heterojunctions of Co_3_O_4_ nanoparticles uniformly anchored to Ti_3_C_2_ MXene using in situ electrostatic assembly and solvothermal methods. The particle size of Co_3_O_4_ anchored to Ti_3_C_2_ MXene was compared with that of pure Co_3_O_4_. It was found that the presence of Ti_3_C_2_ MXene played an important role in the particle size of Co_3_O_4_ during the solvothermal process. In the electrocatalytic process, MXene helps to promote the contact between Co_3_O_4_ and the electrolyte because of its unique hydrophilic properties, and its own metal conductivity is conducive to accelerating the electron transfer of Co_3_O_4_ in the OER process, and the heterogeneous structure can shorten the charge transfer pathway, significantly improving the electrocatalytic activity.

Furthermore, in situ growth of Co_3_O_4_ onto two-dimensional materials has been explored; the deposition of Co_3_O_4_ on a carrier of Ag@B has also been investigated. The incorporation of Ag nanoparticles as a conductive material can improve the conductivity of Co_3_O_4_ and promote electron transfer during the oxidation process of Co^3+^. Moreover, the formation of B–Co and B–O bonds between B and Co_3_O_4_ can lower the energy barrier in the OER process, leading to improved efficiency of OER [111]. In addition, deposition of a conductive coating on the surface of Co_3_O_4_ is another way to improve the conductivity of the catalyst [112,113]. In line with this, Tong et al. [114] prepared Co_3_O_4_/PPy (polypyrrole) by electrodepositing PPy onto the surface of Co_3_O_4_. Co_3_O_4_/PPy exhibits a very low overpotential (220 mV vs. 320 mV) compared with pure Co_3_O_4_ at the current density of 10 mA cm^−2^, and only 310 mV can reach a current density of 100 mA cm^−2^. The results suggest that the deposition of a conductive polymer, such as PPy, can enhance the conductivity of Co_3_O_4_ and improve the catalytic performance of the material for OER.

## 3. Intrinsic Activity Enhancement

It is crucial to enhance the intrinsic activity of Co_3_O_4_-based catalysts, which involves increasing the catalytic efficiency of OER at each active site and improving the coordination between them, even under the same number of active sites. To achieve this, two common mechanisms have been identified: the defect effect [115,116] and the synergistic effect [117,118]. These mechanisms aim to increase the intrinsic activity of the catalyst by improving its structure and properties, rather than just increasing the active surface area or material transport channel, which are important but not sufficient factors for optimal catalytic performance. Therefore, a comprehensive approach that considers both external and intrinsic factors is imperative to achieve the highest possible catalytic performance.

### 3.1. Defect Effect

In the preparation process of a catalyst, the occurrence of defects will cause significant changes to chemical composition, strain, atomic/ionic coordination, and so on, which might be conducive to the catalysis of OER. Therefore, the defect effect has become one of the basic concepts of rational catalyst design [115,116,119].

As one of the simplest and convenient methods to realize the defect effect of transition metal oxide-based catalysts [120], oxygen vacancy defect engineering is also applicable to Co_3_O_4_. He et al. [121] demonstrated an MOF-to-MOF-based self-template strategy to fabricate Fe-doped Co_3_O_4_ hollow nanosheets with rich oxygen vacancies. The hollow nanosheets provide a large active surface area and a short mass/electron diffusion pathway, and the Fe doping induces the formation of oxygen vacancies, which further adjust the electronic structure and improve the conductivity. And Cai et al. [122] prepared Co_3_O_4_ single-crystal nanosheets rich in oxygen vacancies using a mild solvothermal method. XPS results revealed high concentrations of O-vacancy defects in Co_3_O_4_ nanocrystals, leading to many suspended bonds and surface unsaturation, which reduced energy and improved catalyst stability [123]. Theoretical calculations showed that the introduction of oxygen vacancies exposed the Co site to the electrolyte, increased the active Co^2+^ and electron concentration around the metal Co site, and reduced the activation energy of the reaction, resulting in improved intrinsic activity. This conclusion was consistent with the findings of Huang et al.’s [33] characterization study of Co_3_O_4_ defects. In fact, many synthesis methods of oxygen-rich Co_3_O_4_ cannot control the concentration of oxygen vacancy, so acid treatment is one of the promising means to produce oxygen vacancy. H^+^ can capture O^2−^ in transition metal oxides (TMOs). Therefore, Co_3_O_4_ with different oxygen vacancy concentrations can be obtained by soaking the synthesized Co_3_O_4_ in 6 M hydrochloric acid (HCl) solution for different times and then washing and drying under vacuum conditions. However, it is worth noting that more time of acid treatment is not better. Too long a treatment time may affect the structure of Co_3_O_4_ and cause it to collapse, resulting in partial shielding of catalytic sites [124]. Although the catalytic performance of the catalyst in this experiment is not that excellent, the novel preparation method proposed by it is hopeful to provide ideas for the preparation of other Co_3_O_4_-based materials.

**Figure 7:**
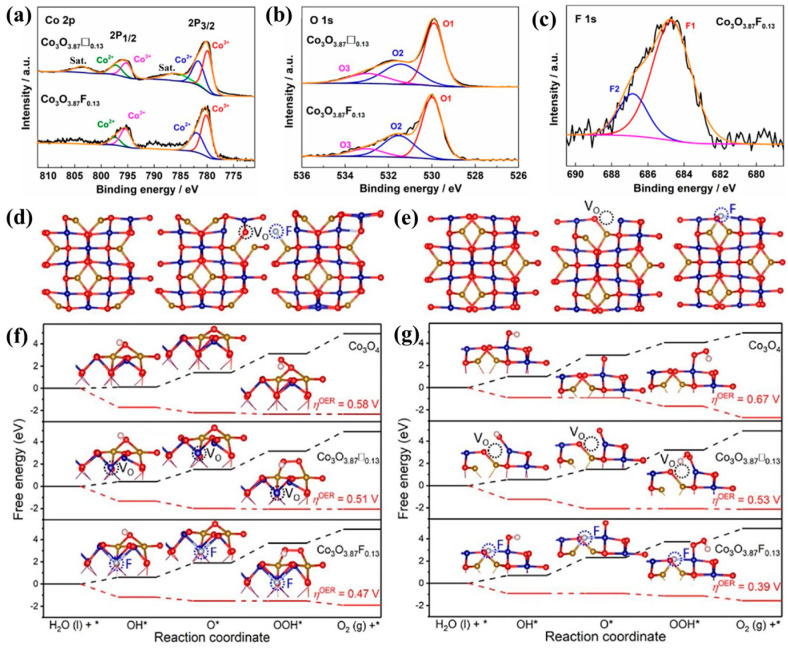
High-resolution (**a**) Co 2p, (**b**) O 1s XPS spectra of Co_3_O_3.87_◻_0.13_ and Co_3.87_F_0.13_, (**c**) F 1s XPS spectra of Co_3_O_3.87_F_0.13_. (**d**,**e**) Side views of optimized geometries of (110)-A and (110)-B surfaces for Co_3_O_4_, Co_3_O_3.87_◻_0.13_, and Co_3_O_3.87_F_0.13_, respectively. The O vacancy and doped F atom are marked by black and blue circles, respectively. (**f**,**g**) The Gibbs free energy diagram of the OER process on (110)-A and (110)-B surfaces for Co_3_O_4_, Co_3_O_3.87_◻_0.13_, and Co_3_O_3.87_F_0.13_, respectively. (Inserts show the top views of corresponding structures of the intermediate adsorbates. The O atoms are in red, Co^2+^ is gold, Co^3+^ is blue, H is purple, and F is silver. η^OER^ means the difference between the measured and the thermodynamically predicted potential for each half reactions.) (Reproduced from ref. [125] with permission from Elsevier).

The presence of oxygen vacancies will make the processing of OH^−^ at the catalytic active site smoother. Therefore, some researchers proposed the phase method of replacing oxygen vacancies with elements that are more electronegative than oxygen. Zeng et al. [125] synthesized mesoporous defective Co_3_O_4−x_ by calcining a silica hard-template (SBA-15) and used the fluorine element to replace the oxygen vacancy in Co_3_O_3.87_◻_0.13_ (◻ represents oxygen vacancies) to obtain Co_3_O_3. 87_F_0.13_. The XPS results show that both oxygen vacancies and fluorine substitution can change the electronic structure of Co, reduce Co^3+^ to Co^2+^, and increase the ratio of Co^2+^ to Co^3+^ (Figure 7a–c). By comparing the catalytic properties of Co_3_O_3.87_◻_0.13_, Co_3_O_3.87_F_0.13_, and pure Co_3_O_4_, it was found that the OER catalytic performances of both Co_3_O_3.87_◻_0.13_ and Co_3_O_3.87_F_0.13_ were improved. Moreover, the OER catalytic performance of fluorine-substituted Co_3_O_4_ (Co_3_O_3.87_F_0.13_) is slightly better than that of Co_3_O_4_ with oxygen vacancies (Co_3_O_3. 87_◻_0.13_). This is because the introduction of fluorine makes the d-band center of the Co 3d orbit drop more, and the ability of Co to reduce electron richness increases. The generation of oxygen vacancy does not change the rate-limiting step of OER in Co_3_O_4_ but only reduces the overpotential in the rate-limiting step. However, the introduction of fluorine changes the rate-limiting step of the catalyst from OOH* → O_2_ to O* → OOH*, leading to lower overpotential; thus, the catalytic performance of Co_3_O_3.87_ F_0.13_ is better than that of Co_3_O_3.87_◻_0.13_ (Figure 7d–f). Therefore, element substitution for oxygen vacancies is a promising solution to improve the OER performance of Co_3_O_4_-based catalysts. On this basis, a method of producing Co_3_O_4_ with oxygen vacancy and fluorine dual-defect has been proposed and achieved good results [126]. In addition to fluorine, we believe that there might be more elements that could replace oxygen vacancies, such as Cl, Br, and I in the same group as fluorine, and S, Se, and Te in the same group as oxygen. Due to the unique physical and chemical properties of each element, these elements might become the development direction of future research and have certain potential. For example, CoS_2_ itself has good OER catalytic performance. If S could replace part of the oxygen vacancies, the energy band might be further reduced, resulting in comprehensive performance of CoS_2_ and Co_3_O_4_.

**Figure 8:**
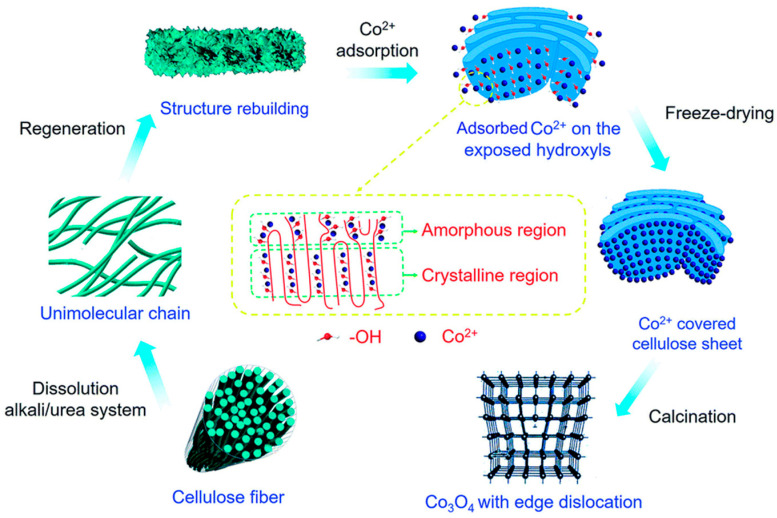
Schematic diagram of DA-Co_3_O_4_ synthesis with the regenerated cellulose template (Reproduced from ref. [128] with permission from RSC).

**Figure 9:**
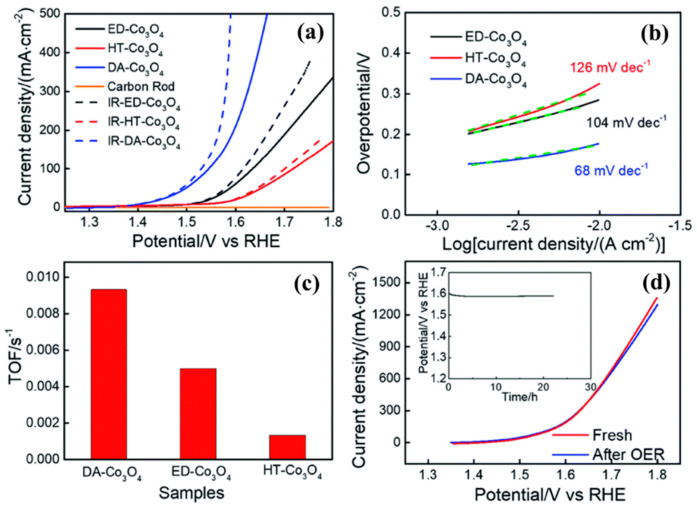
The comparison of the DA-Co_3_O_4_-coated electrode with ED-Co_3_O_4_ and HT-Co_3_O_4_-coated electrodes and pure carbon rod electrodes, and the corresponding imaginary lines are IR-corrected polarization curves (**a**); Tafel slopes of DA-Co_3_O_4_, ED-Co_3_O_4_, and HT-Co_3_O_4_-coated electrodes (**b**); TOFs of DA-Co_3_O_4_, ED-Co_3_O_4_, and HT-Co_3_O_4_-coated electrodes, which were calculated from the response current at η = 300 mV (**c**); polarization curves of the DA-Co_3_O_4_-coated electrode before and after 24 h electrolysis. Inset: time dependence of current density for the DA-Co_3_O_4_-coated electrodes in a 1 M KOH electrolyte (**d**). (Reproduced from ref. [128] with permission from RSC).

The vacancy defects that belong to point defects can improve the performance of catalysts [42]. Moreover, the dislocation, as one of the types of line defects, might also have a positive impact on the performance of catalysts [42,127]. Li et al. [128] utilized a template method to adsorb Co onto regenerated cellulose and then calcined it to obtain a defect-activated Co_3_O_4_ nanosheet catalyst. During the calcination process, the decomposition of cellulose played a critical role in the production of Co_3_O_4_. The presence of dislocations altered the electronic structure and chemical properties of the catalyst, leading to distortion of the Co-O bond in the material, which improved its intrinsic activity, enhanced the adsorption/desorption ability of charge and reactants, and thus facilitated their transfer (as shown in Figure 8). The defects generated by the edge increase the exposure of the active site of the catalyst, and the {011} crystal surface makes the edge of the Co_3_O_4_ nanosheet partly metallized, which improves the conductivity of the catalyst itself. All the above factors contribute to the super-strong catalytic performance of this catalyst. According to the electrochemical testing results, it only needs an overpotential of 183 mV to transport a current density of 10 mA cm^−2^, and the catalytic current density reached 500 mA cm^−2^ under the voltage of less than 1.6 V by the IR compensated curve (Figure 9a–d). This result is striking for a Co_3_O_4_-based catalyst. However, it is a pity that the preparation process is somewhat complicated, and with some improvements, it might support industrial production. In addition to dislocations, defects arising from tensile strain also affect the catalysis performance of OER. For example, when Cr is doped into Co_3_O_4_, tensile strain is generated, and then Cr is corroded with lye, resulting in the previously generated tensile strain being retained even with the loss of some of the Cr. This strain reduces the width of the d-band, causing the center of the d-band to shift, which improves the adsorption efficiency of the OER intermediate substance [129].

Most defect studies to date have focused on point defects, such as oxygen vacancy defects, and line defects, such as dislocation defects. However, the other two types, surface defects and bulk defects, are relatively less studied. Perhaps in future studies, surface defects will also become one of the focuses of defect research, and defect types will be integrated to further increase the performance of catalysts.

### 3.2. Synergistic Effect

In the field of catalysis, a synergistic effect refers to the enhancement of catalytic performance through adding other components to a catalyst during the synthesis process, such that the combined effect of multiple components is greater than the sum of their individual effects [130].

The use of Co_3_O_4_-based catalysts provides an opportunity to improve catalytic performance through the incorporation of additional components. Zhang et al. [131] synthesized a self-assembled hybrid SrCo_0.55_Fe_0.5_O_3−δ_ nanorod composed of edge-sharing Co_3_O_4_ and corner-sharing SrCo_0.5_Fe_0.5_O_3−δ_ by Co-site enrichment. The edge-sharing structure not only reduces the steric resistance but also improves its structural stability, and the existence of the corner-sharing structure enhances its electron transport ability. Apart from this, Ahmed et al. [132] embedded Co_3_O_4_ nanorods modified with MoO_3_ by a simple hydrothermal method and calcined conductive g-C_3_N_4_ to form a hybrid structure of Co_3_O_4_/MoO_3_/g-C_3_N_4_. The transmission electron microscopy (TEM) images showed that MoO_3_ particles were successfully attached to the surface of Co_3_O_4_ and formed a heterojunction interface. Furthermore, the formation of Co_3_O_4_/MoO_3_/g-C_3_N_4_ composite structure was confirmed by attenuated reflectance-Fourier transform infrared (ATR-FTIR) spectra. In addition to the existence of g-C_3_N_4_ as an excellent conductive substrate, the advantage of the composite structure also depends on the modulation effect of Mo on Co_3_O_4_. The Co_3_O_4_/MoO_3_ synergy allows the Co–O–Mo bond to function as the electron transfer channel and the active sites to improve the reaction kinetics. Therefore, this structure only requires 206 mV to drive the current density of 10 mA cm^−2^ in 1 M KOH solution, which is better than most Co_3_O_4_-based catalysts. Compared with pure Co_3_O_4_, the overpotential is reduced by nearly 250 mV. Meanwhile, the Tafel slope of Co_3_O_4_/MoO_3_/g-C_3_N_4_ was only 60 mV dec^−1^, which belongs to the low level among Co_3_O_4_-based catalysts. In fact, besides MoO_3_ mentioned above, Dai et al. [133] demonstrated a synergistic effect between Co_3_O_4_ and transition metal oxides. Porous CuMoO_4_@Co_3_O_4_/NF nanosheets were prepared using a simple hydrothermal and impregnation method. Interestingly, because Co_3_O_4_ can be used as an inducer in the preparation process to transform Cu^+^ to Cu^2+^, compared with CuMoO_4_/NF, the ratio of Cu^2+^/Cu^+^ in CuMoO_4_@Co_3_O_4_/NF is higher, which promotes the contact between OH^−^ and the catalyst and the transportation of O_2_, thus promoting the catalytic efficiency of OER. In the subsequent catalytic performance test, combined with the comparison between CuMoO_4_@Co_3_O_4_ ( η _50_ = 251 mV), CuMoO_4_ ( η _50_ = 363 mV), and Co_3_O_4_ ( η _50_ = 373 mV), it can be clearly known that CuMoO_4_ and Co_3_O_4_ have a catalytic synergistic effect on each other. XPS spectra also confirmed the strong electron interaction between CuMoO_4_ and Co_3_O_4_, resulting in electron transfer and electron coupling. In addition, during the 120 h stability test, CuMoO_4_@Co_3_O_4_/NF shows excellent stability, which might be due to the extremely stable metal–oxygen bond between Cu–O, Mo–O, and Co–O, thus promoting the stability of the whole structure. Phosphorization has been one of the effective means to improve the performance of OER [134,135] because the phosphorization process could improve the number and intrinsic activity of the active sites of the catalysts [78]. Based on this, Alhakemy et al. [136] prepared a Co_3_O_4_-C microrod structure covered by film-like FeMoP on nickel foam by solvothermal, annealing, and phosphating, labelled as Co_3_O_4_-C@FeMoP/NF. The catalyst showed remarkable performance in OER tests ( η _20_ = 200 mV, C_dl_ = 95.3 mF cm^−2^). It was found that the C_dl_ of Co_3_O_4_-C@FeMoP/NF is not as good as the C_dl_ of Co_3_O_4_-C/NF, but the catalytic performance is much better. Therefore, the increase of active sites is not the only factor that improves the catalytic performance of Co_3_O_4_-C@FeMoP/NF. The normalized curve also proved that the improvement of catalytic activity of the active sites themselves was the main reason for improving the catalytic efficiency of OER. In addition to improving the electron transmission level of Co_3_O_4_-C due to the certain conductivity of phosphating FeMoP, the synergistic effect between Co_3_O_4_-C and FeMoP is an important reason for improving the catalytic level. Other than transition metal oxides/phosphates, the heterogeneous structure between Co_3_O_4_ and transition metal sulfide MoS_2_ will produce significant differences in electronegativity between S and O, which can improve the conductivity and charge transfer rate, and the synergistic effect of the two makes the active sites of the catalyst more efficient in adsorption and resolution of intermediates in the OER process, thus improving the activity of OER [137].

**Figure 10:**
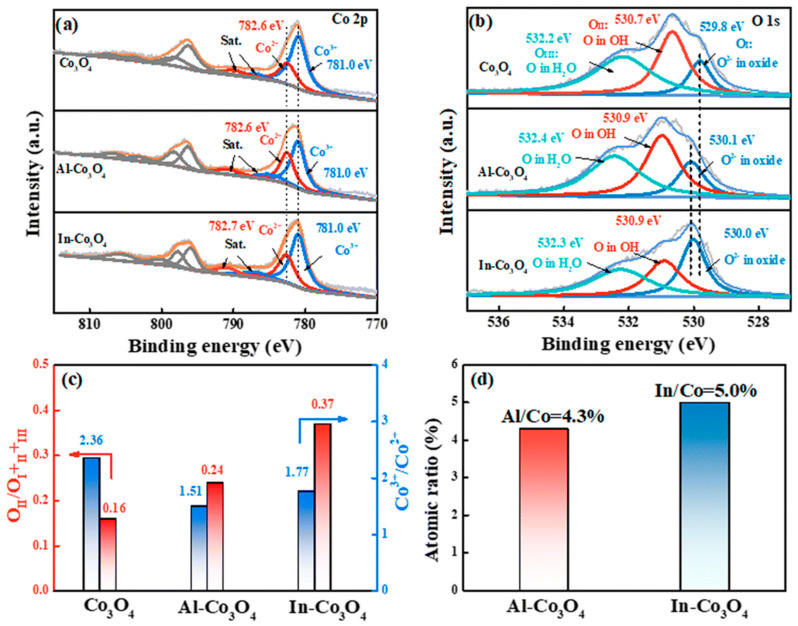
The high-resolution XPS spectra of (**a**) Co 2p and (**b**) O 1s for Co_3_O_4_, Al-Co_3_O_4_, and In-Co_3_O_4_. (**c**) The Co^3+^/Co^2+^ and O_II_/O_I+II+III_ atomic ratios from the XPS results. (**d**) The ratios of Al/Co and In/Co in Al-Co_3_O_4_ and In-Co_3_O_4_ from the EDS results (Reproduced from ref. [140] with permission from RSC).

**Figure 11:**
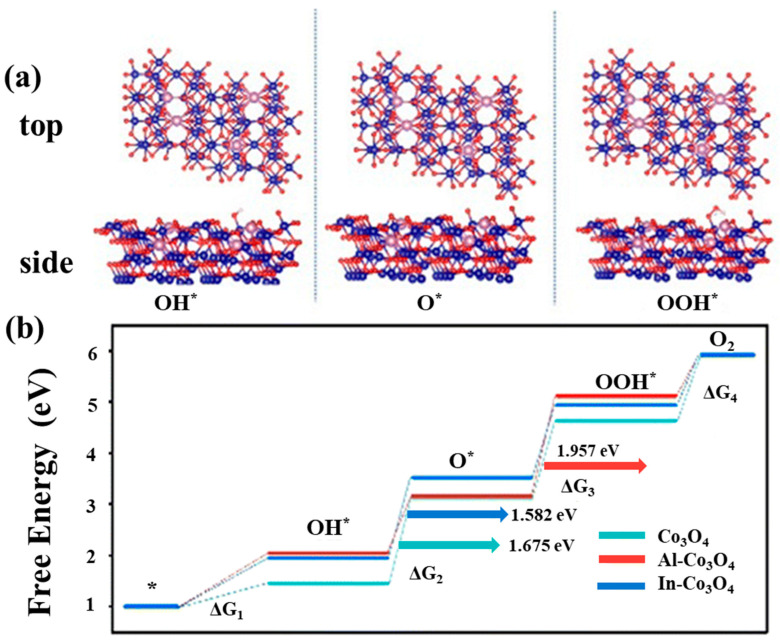
(**a**) The optimized configurations of In-Co_3_O_4_ chemisorption of OH*, O*, and OOH* intermediates. (**b**) The calculated free energy diagrams for the OER on Co_3_O_4_, Al-Co_3_O_4_, and In-Co_3_O_4_ at applied potentials of 0 V (Reproduced from ref. [140] with permission from RSC).

**Figure 12:**
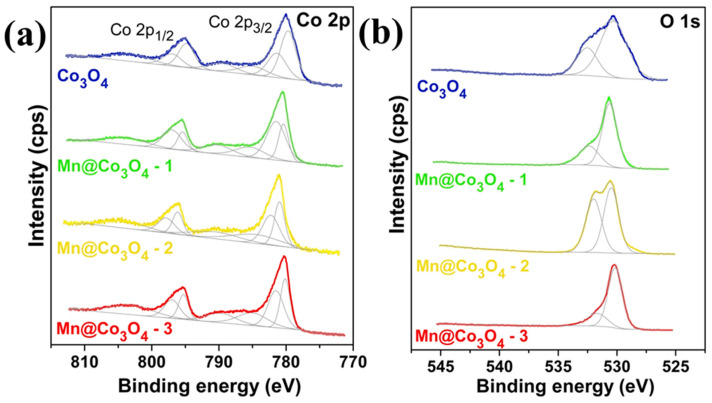
High-resolution XPS spectra of the studied catalysts. (**a**): Co 2p and (**b**): O 1s. (Reproduced from ref. [141] with permission from RSC).

**Figure 13:**
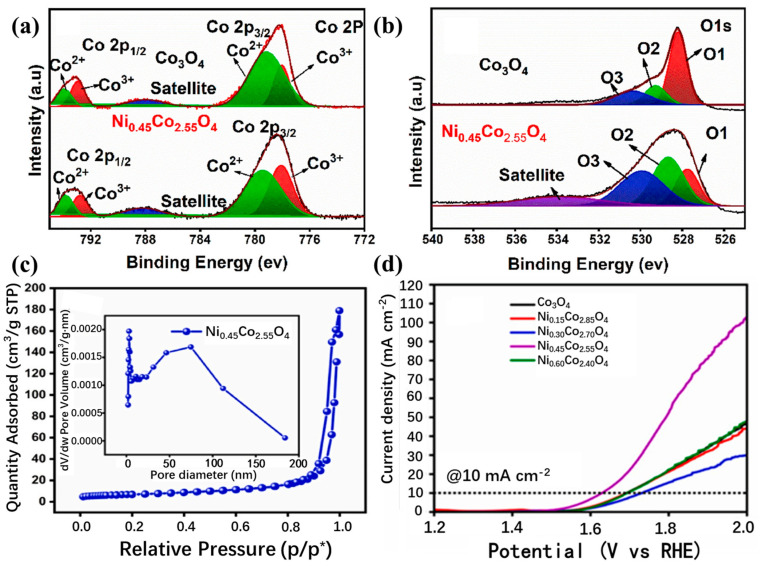
High-resolution XPS spectra of Co 2p (**a**) and O 1s (**b**) of the Co_3_O_4_ and Ni_0.45_Co_2.55_O_4_ compounds. The nitrogen absorption-desorption plots of (**c**) Ni_0.45_Co_2.55_O_4_ with the inset BJH desorption pore volume data. (**d**) LSV curves of Ni_x_Co_3−x_O_4_ catalysts in 1 M KOH electrolyte with a sweep rate of 5 mV s^−1^. (Reproduced from ref. [142] with permission from Elsevier).

**Figure 14:**
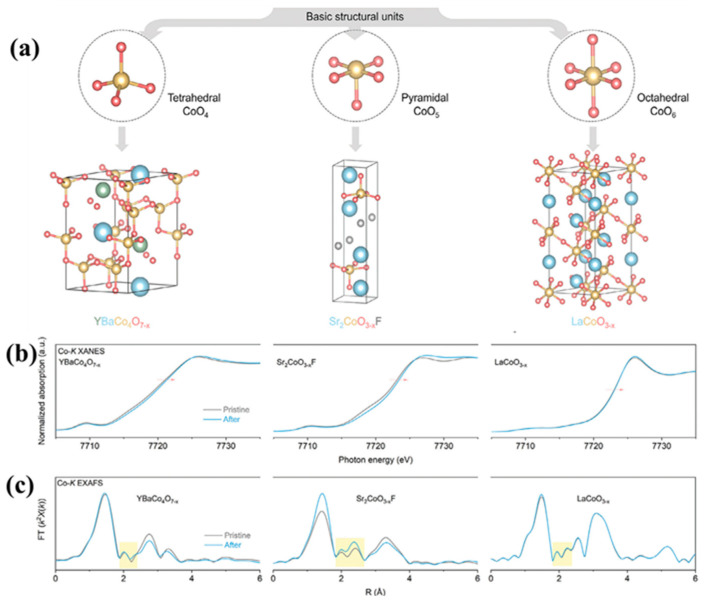
(**a**) Structures of model coordinated oxides YBaCo_4_O_7−x_, Sr_2_CoO_3−x_F, and LaCoO_3−x_ made up of tetrahedral CoO_4_, pyramidal CoO_5_, and octahedral CoO_6_ ligands, respectively. Reconstruction of bulk local structure. (**b**) Co-*K* XANES spectra before and after OER for model oxides. (**c**) Co-*K* EXAFS spectra before and after OER for model oxides. (Reproduced from ref. [144] with permission from Wiley).

It is not only the heterogeneous structure but also the doping at the atomic level that can trigger the synergistic effect of the catalyst [47,138], especially in Co_3_O_4_, where the possibility of synergistic effect is greatly increased due to the presence of tetrahedral Co^2+^ and octahedral Co^3+^ [39,139]. Based on the above possibilities, Shao et al. [140] reported a simple synthesis method using the co-precipitation method for selective doping of Co_3_O_4_ and tested the doping effects of Al and In, respectively. XPS spectra show that the Co 2p peaks at 781.0 eV and 782.6 eV correspond to the Co^3+^ and Co^2+^ species. In In-Co_3_O_4_, the Co^2+^ peaks change to higher binding energies, indicating the effect of In doping. O 1s spectra show the contributions of oxides (O_I_), hydroxides (O_II_), and adsorbed oxygen (O_III_). And the proportion of O_II_ in all O increases gradually from Co_3_O_4_, Al-Co_3_O_4_, and In-Co_3_O_4_, which contributes to the improvement of the conductivity of the catalytic materials (Figure 10a–d). However, electrocatalytic results show that the electrochemical performance of Al-doped Co_3_O_4_ decreases instead of increasing, which might be due to the partial substitution of Co^3+^ by Al, resulting in a decrease in the number of active sites. In addition, In-Co_3_O_4_ can retain the activity of octahedral position points of Co^3+^ while activating Co^2+^ tetrahedral sites. Therefore, its catalytic activity is improved compared with that of pure Co_3_O_4_. The calculation of DFT also shows that the free energy barrier in the conversion of O_2_ by Al-Co_3_O_4_ is large (1.957 eV), while In, which has a synergistic effect with Co_3_O_4_, makes the energy barrier of In-Co_3_O_4_ relatively small (1.582 eV) in each step of the catalytic process, thus providing superior catalytic performance (Figure 11a,b). The synergistic effect caused by the introduction of a single element is not only manifested in changing the ratio of Co^3+^ and Co^2+^, but also in the defect effect of Co_3_O_4_. Silva et al. [141] found that the incorporation of Mn into Co_3_O_4_ changed the crystal structure and electronic properties of Co_3_O_4_. After doping Co_3_O_4_ with Mn, a decrease in Co^3+^ content was observed by XPS analysis, which suggested that the incorporation of Mn may result in partial reduction of Co^3+^ to Co^2+^ (Figure 12a). The O 1s peak of the XPS spectrum consists of two distinct contributions, with a low binding energy peak indicating a metal–O bond and a high binding energy peak indicating the presence of surface oxygen vacancies (Figure 12b). These changes directly improve the reduction and oxidation capacity and OER performance of the Co_3_O_4_ catalyst. And this has been confirmed by Murugesan et al. [142]. After incorporating Ni into Co_3_O_4_, the Co 2p 3/2 and 2p 1/2 peaks and the high binding energy peak of oxygen deficiency demonstrate the presence of oxygen vacancies, indicating that Ni doping is beneficial for OER enhancement (Figure 13a,b). In addition, Ni doping will reduce the grain size and form nanoparticles with a large specific surface area and large pore size (Ni_0.45_Co_2.55_O_4_) at a certain content, which is conducive to mass transport and improves catalytic reaction activity (Figure 13c,d). According to the above results, some atoms can replace the tetrahedral or octahedral sites of Co_3_O_4_ to change the ratio of Co^2+^/Co^3+^, thus effectively enhancing the catalytic effect of OER. Guo et al.’s [38] research has verified this view. The authors prepared an Ru- and Ni-co-doped Co_3_O_4_ catalyst by a simple hydrothermal method, in which the introduction of Ru replaced part of the Co^3+^ octahedral position points so that the activity of some octahedral position points is better, and the introduction of Ni^3+^ reduced the energy barrier of electron transfer of Co_3_O_4_ during the adsorption of OH surface intermediates and increased certain oxygen vacancies. The synergistic effect between the two elements and Co_3_O_4_ greatly improves the catalytic efficiency of OER. Besides the above Ru–Ni dual-doping can effectively generate synergistic effects with Co_3_O_4_; for example, Mn and S can also be used as dual-doping elements [143]. However, different from the former research, the introduction of Mn and S changes the proportion of different valence states of the Co element not by replacing the tetrahedron or octahedron, but by changing the electronic structure of the center of the Co species. The introduction of Mn and S changed the electronic structure of Co atoms and increased the free electrons that could be used for catalysis. Moreover, the doping of S increased the intrinsic conductivity of the catalyst, thus showing satisfactory performance in the subsequent OER test. In order to better understand the influence of the structure of Co_3_O_4_ on the catalyst, Guan et al. [144] not only analyzed the mechanism of tetrahedral coordination (CoO_4_) and octahedral coordination (CoO_6_) structures but also investigated the pyramidal (CoO_5_) coordination structure using advanced operando characterizations combined with systematic computations. As shown in Figure 14a, the configuration of CoO_5_ is asymmetric, which is the most active coordination environment in OER. CoO_6_ presents a regular and symmetrical octahedral structure, which exhibits high stability. However, CoO_4_ shows moderate catalytic activity due to high symmetry. The interaction between Co atoms and OH^−^ is significantly affected by its local coordination environment. Specifically, the CoO_4_ site exhibits moderate reactivity and partial surface remodeling, and CoO_6_ is structurally stable but less active. However, due to its unsaturation, the pyramidal CoO_5_ coordination can be remodeled into highly active amorphous CoOOHₓ during the OER process, and the corresponding activity is enhanced, but the stability is reduced (see Figure 14b,c). Therefore, the manipulation of local Co–O configurations is regarded as a key strategy to improve the performance of spinel-based catalysts.

To summarize the catalytic performance of various electrocatalysts discussed in Section 2 and Section 3, Table 2 provides an overview of the catalysts, electrode types, operational conditions, and the corresponding current densities achieved under different experimental setups.

## 4. Conclusions and Outlook

This paper presents a systematic introduction of the internal mechanisms and preparation strategies for Co_3_O_4_-based catalysts used in OER. To improve the internal and external activity of the catalyst, morphological engineering and catalyst anchoring are commonly employed to increase the active surface area or the number of active sites. Morphology engineering, including the design of nanostructures such as 1D, 2D, or 3D nanomaterials, enhances the exposure of active sites, contributing to better OER performance. Moreover, integrating the catalyst with conductive materials improves the material transport channels during OER, boosting external catalytic performance. To enhance the intrinsic activity of Co_3_O_4_-based catalysts, defect engineering, including oxygen vacancies and dislocations, along with synergistic effects from doping or substituting with other elements, is typically used to optimize the catalyst’s efficiency. Therefore, the synergy between morphological engineering and intrinsic structural modifications (e.g., defect and doping effects) offers a promising path for improving the OER performance of Co_3_O_4_-based catalysts under alkaline conditions. Recent years have seen increasing interest in Co_3_O_4_-based catalysts due to their high stability, multiple active sites, simple preparation methods, and good compatibility, making them a focal point in alkaline OER research. Despite significant progress in improving catalytic performance, there are still urgent challenges that need to be addressed.

(1) Establish a set of standard catalytic evaluation systems.

For example, many studies use NF as a control for comparing catalytic performance, but the results of the catalytic performance of most NF are not the same, especially in the results dominated by the LSV curve. The overpotential difference of NF under the same current density can reach tens of mV, even more than 100 mV, which might be due to the influence of the manufacturers’ preparation procedures and electrochemical testing instruments. Theoretically, there should be no significant difference in the catalytic performance of pure NF, and the difference in the catalytic performance of Co_3_O_4_-based catalysts is reflected in different papers, which reduces the credibility of the catalytic performance of Co_3_O_4_-based catalysts. In addition, an electric potential with a geometric surface area normalization on the steady-state current density cannot be used as an active parameter because the loading of the catalyst is not taken into account. For example, samples of the same material can show different potentials at a defined mA cm_geo_^−2^ depending on the amount of catalyst used. Therefore, it is especially important to establish a set of standard catalytic evaluation systems, not only for Co_3_O_4_-based catalysts; in fact, for all catalysts, a uniform evaluation standard is conducive to evaluating the real catalytic performance. For instance, if all OER studies were controlled with a nickel foam of fixed length, width, and thickness, and the difference in performance with other prepared catalysts was one of the criteria, it would be more convincing than simply subtracting 1.23 V from the potential. In addition, there are different test times and standards for catalytic stability, such as 10 mA cm^−2^/100 mA cm^−2^, 10,000 s/100,000 s/24 h/120 h, etc., which might cause trouble when comparing the stability of different catalysts. Therefore, it is important to establish a unified standard catalytic performance evaluation system for the follow-up development of catalysis.

(2) Integrating favorable factors.

In recent years, Co_3_O_4_-based catalysts have been extensively studied to coordinate the combination of various favorable factors to improve the OER performance. However, it is important to note that each factor has a unique influence on catalytic performance. For instance, morphology engineering has been investigated to enhance the active sites and active surface areas of the catalyst. Nevertheless, different morphologies of Co_3_O_4_ might lead to distinct effects on catalysis, thus requiring further research to identify the optimal morphology group. Despite the diversity of morphologies available for Co_3_O_4_-based catalysts, the underlying mechanism of their formation has not been fully explored, which could hinder researchers from obtaining the desired morphology while attempting to integrate other favorable factors. Alteration of synthesis temperature, solvent, raw materials, or employment of high-temperature treatment methods might influence catalytic activity, but the combination of these individual factors might not achieve the desired effect, which presents a challenge in integrating multiple favorable factors.

(3) Industrialization synthesis of the catalyst.

In material science, it is essential to focus on the practical applications of catalytic water electrolysis. In most cases, the preparation of catalysts involves coating the catalyst particles on the electrode or growing them in situ on the substrate. The method performs well at laboratory scale; however, the in situ growth of catalysts does not have the potential for industrialization due to its excessive cost in terms of manpower and material resources, and its repeatability is relatively low. Even with the current state of science and technology, it is challenging to develop the conditions required for the large-scale production of in situ growth catalysts. On the other hand, preparing the catalyst as a particle is the best approach, but additional components are required to bind the catalyst to the electrode, making it a more complex process. From our point of view, the most promising approach for water electrolysis catalysts is direct existence in the electrolyte or self-adsorption on the electrode, offering excellent catalytic performance. Although there are limited studies in this area, this approach has the potential to revolutionize industrial water electrolysis and significantly reduce costs. Furthermore, current catalysts lack stability, failing to meet industrial standards, and requiring replacement after a period of use, which appears to be a substantial challenge to cost-effectiveness.

(4) Better understanding of the OER mechanism of Co_3_O_4_-based catalyst.

Although plenty of studies regarding the mechanism of OER under alkaline conditions have been reported in recent years, the mechanism of Co_3_O_4_ in OER has not been thoroughly explained. OER is a multi-step process, and the Co^2+^ and Co^3+^ active sites in Co_3_O_4_ might play different leading roles in different steps. Otherwise, it is difficult to explain the high ratio of Co^2+^/Co^3+^ in some catalysts while the low ratio of Co^2+^/Co^3+^ in some other catalysts, but all of them might have good catalytic performance. In addition, many studies have shown that Co_3_O_4_-based catalysts undergo structural restructuring in the process of catalysis, and the actual active matter is high-priced oxides or hydroxides. Therefore, it is particularly important to thoroughly understand the reaction mechanism, structural changes, and active centers of Co_3_O_4_-based catalysts in the process of OER. In situ characterization might resolve this problem in the future, which can find the surface valence state of the Co_3_O_4_-based catalyst and provide a solid theoretical basis for the preparation of catalysts.

(5) Challenges and opportunities for practical applications of Co_3_O_4_-based catalysts.

In practical water-splitting applications, the key challenge to achieving high current density and long-term stable operation is developing high-performance electrocatalysts. Although Co_3_O_4_-based electrocatalysts have shown promising potential in terms of activity and adjustability, further validation is needed for application under industrial-scale conditions, such as >500 mA·cm^−2^. Therefore, future research should focus on the long-term stability of intrinsic activity in strong alkali or acid environments while improving its intrinsic activity. In addition, innovations such as electric field treatment and composite engineering (e.g., Co_3_O_4_–Fe_2_O_3_, Ni/Co_3_O_4_) have significantly improved conductivity and durability, demonstrating their applicability in the real world, which will enable the integration of Co_3_O_4_-based catalysts into membrane electrode assemblies (MEAs) and, in combination with renewable energy systems such as photovoltaics and wind energy, improve the feasibility of integrating Co_3_O_4_-based catalysts into MEAs.

## Figures and Tables

**Table 1:** Comparison of the structures and performances of Co_3_O_4_-based catalysts and commercial catalysts.

Catalyst	Morphology	*j* (mA cm^−2^)	*η* (mV)	Electrolyte	Ref.
Co_3_O_4_@Ti	Nanoneedles	20	416	1 M KOH	[57]
Ti@M-Co_3_O_4_	Nanoneedles	10	450	0.1 M KOH	[77]
NiO_x_@Co_3_O_4_/CC	Nanowire	10	360	0.1 M KOH	[78]
Ru-Co_3_O_4_/CoP/TM	Nanowire	10	293	1 M KOH	[78]
Ultrathin Co_3_O_4_ nanofilm	Nanofilms	40	461	1 M KOH	[79]
{112} high-index faceted porous Co_3_O_4_	Nanofilms	10	318	1 M KOH	[70]
B-Co_3_O_4_@ZIF-67	Nanocages	10	334	1 M KOH	[80]
Co-CNT@COF-Pyr	Nanocages	10	438	1 M KOH	[65]
Co_3_O_4_-Ov	Sea urchin-shaped structures	20	280	1 M KOH	[81]
Co_3_O_4_/NF	Sea urchin-shaped structures	20	327	1 M KOH	[71]
Co_3_O_4_ nanosheets	Flower-shaped structures	10	380	0.1 M KOH	[82]
CoCe HNF	Flower-shaped structures	10	315	1 M KOH	[72]
Co_3_O_4_|CoP	Core-shell structure	10	320	1 M KOH	[66]
Co_3_O_4_@NiCo LDH	Core-shell structure	15	279	1 M KOH	[73]
Ni_3_S_2_@MoO_3_@Co_3_O_4_@AMO/NF	Core-shell structure	10	248	1 M KOH	[83]
Ru–CoPO	Nanowire	10	310	1 M KOH	[84]
Au–IrO_2_	Flower-shaped structures	10	286	0.1 M KOH	[85]

**Table 2:** Summary of catalyst performance in OER.

Catalyst	Electrode Type (Work, Counter, and Reference Electrodes)	*j* (mA cm^−2^)	*η* (mV)	Electrolyte	Ref.
Mn@Co_3_O_4_	Mn@Co_3_O_4_, platinum bar, and Ag|AgCl electrode (3 mol L^−1^ KCl)	10	320	1 M KOH	[145]
Hybrid-phase SrCo_0.55_Fe_0.5_O_3−δ_	Hybrid-phase SrCo_0.55_Fe_0.5_O_3−δ_, graphite rod, and Ag/AgCl	10	290	1 M KOH	[131]
V_o_-Fe–Co_3_O_4_	V_o_-Fe–Co_3_O_4_, Pt wire, and Hg/HgO	10	231	1 M KOH	[121]
Co_3_O_4_/PPy-120	Co_3_O_4_/PPy-120, graphite rod, and Ag/AgCl	10	140	1 M KOH	[114]
M-Co_3_O_4_/NPC	Co_3_O_4_/NPC, platinum foil, and reversible hydrogen electrode (RHE)	10	302	1 M KOH	[103]
Ir_0.33_@Co_3_O_4_	Glassy carbon, Pt mesh, and Ag/AgCl	10	296	1 M KOH	[88]
In-Co_3_O_4_	In-Co_3_O_4_, platinum slice, and Hg/HgO	10	340	1 M KOH	[140]
Co_3_O_3.87_◻_0.13_	Co_3_O_3.87_◻_0.13_, Pt wire equipped with isolation tube, and Hg/HgO	10	440	0.1 M KOH	[125]
Co_3_O_4_ NS/NF	Co_3_O_4_ NS/NF, Pt foil, and saturated Ag/AgCl (3 M KCl) electrode	10	190	0.1 M KOH	[91]
