# Correction: Liu et al. Recent Development on the Synthesis Strategies and Mechanisms of Co_3_O_4_-Based Electrocatalysts for Oxygen Evolution Reaction: A Review. *Molecules* 2025, *30*, 3238

**DOI:** 10.3390/molecules30183692

**Published:** 2025-09-11

**Authors:** Yu Liu, Yifan Jia, Hongxing Jia, Liangjuan Gao

**Affiliations:** 1 College of Materials Science and Engineering, Sichuan University, Chengdu 610065, China; liuyu@alu.scu.edu.cn (Y.L.); jiayifan123@stu.scu.edu.cn (Y.J.); 2 College of Materials Science and Engineering, National Engineering Research Center for Magnesium Alloys, Chongqing University, Chongqing 400044, China

**Addition of an Author**

The authors wish to add one author, Mr. Yu Liu, who made a significant contribution to the original paper [1]. The updated authorship order is Mr. Yu Liu, Mr. Yifan Jia, Dr. Hongxing Jia and Dr. Liangjuan Gao. And the Author Contributions Section should be updated as below.

## Notes

The authors state that the scientific conclusions are unaffected. This correction was approved by the Academic Editor. The original publication has also been updated.
